# Growth Hormone Regulates the Balance Between Bone Formation and Bone Marrow Adiposity

**DOI:** 10.1359/jbmr.091015

**Published:** 2009-10-12

**Authors:** Philip J Menagh, Russell T Turner, Donald B Jump, Carmen P Wong, Malcolm B Lowry, Shoshana Yakar, Clifford J Rosen, Urszula T Iwaniec

**Affiliations:** 1 Department of Nutrition and Exercise Sciences, Oregon State UniversityCorvallis, OR, USA; 2 Linus Pauling Institute, Oregon State UniversityCorvallis, OR, USA; 3 Department of Microbiology, Oregon State UniversityCorvallis, OR, USA; 4 Division of Endocrinology, Diabetes and Bone Diseases, Department of Medicine, Mount Sinai School of MedicineNew York, NY, USA; 5 Maine Center for Osteoporosis Research and Education, St. Joseph HospitalBangor, ME, USA; 6 The Jackson LaboratoryBar Harbor, ME, USA

**Keywords:** osteoblasts, adipocytes, IGF-1, estrogen, parathyroid hormone

## Abstract

Cancellous bone decreases and bone marrow fat content increases with age. Osteoblasts and adipocytes are derived from a common precursor, and growth hormone (GH), a key hormone in integration of energy metabolism, regulates the differentiation and function of both cell lineages. Since an age-related decline in GH is associated with bone loss, we investigated the relationship between GH and bone marrow adiposity in hypophysectomized (HYPOX) rats and in mice with defects in GH signaling. HYPOX dramatically reduced body weight gain, bone growth and mineralizing perimeter, serum insulin-like growth factor 1 (IGF-1) levels, and mRNA levels for IGF-1 in liver and bone. Despite reduced body mass and adipocyte precursor pool size, HYPOX resulted in a dramatic increase in bone lipid levels, as reflected by increased bone marrow adiposity and bone triglyceride and cholesterol content. GH replacement normalized bone marrow adiposity and precursor pool size, as well as mineralizing perimeter in HYPOX rats. In contrast, 17β -estradiol, IGF-1, thyroxine, and cortisone were ineffective. Parathyroid hormone (PTH) reversed the inhibitory effects of HYPOX on mineralizing perimeter but had no effect on adiposity. Finally, bone marrow adiposity was increased in mice deficient in GH and IGF-1 but not in mice deficient in serum IGF-1. Taken together, our findings indicate that the reciprocal changes in bone and fat mass in GH signaling-deficient rodents are not directly coupled with one another. Rather, GH enhances adipocyte as well as osteoblast precursor pool size. However, GH increases osteoblast differentiation while suppressing bone marrow lipid accumulation. © 2010 American Society for Bone and Mineral Research

## Introduction

Growth hormone (GH) is an important physiologic regulator of bone growth and in adults may play an equally important role in regulating bone remodeling. Osteoblasts and chondrocytes have receptors for GH, and addition of the hormone to these cells in vitro increases cell proliferation and differentiation.(1) Congenital GH deficiency in humans and laboratory animals results in decreased bone growth and osteopenia.(2,3) GH levels decrease with age, and GH deficiency may contribute to metabolic syndrome,(4) as well as to common metabolic bone diseases, including postmenopausal and senile osteoporosis.(5,6)

Many of the actions of GH on target cells are mediated through locally produced insulin-like growth factor 1 (IGF-1). Global IGF-1 knockout mice have markedly suppressed bone formation and reduced cortical bone mass despite increased GH levels,(7) whereas targeted overexpression of IGF-1 in mouse osteoblasts results in enhanced bone formation.(8) Targeted disruption of IGF-1 reduces bone growth in mice.(9) Furthermore, locally produced IGF-1 is required for the bone anabolic response to parathyroid hormone (PTH).(10)

Although these findings illustrate the important role of locally produced IGF-1 in bone metabolism, equally compelling data support a role for circulating IGF-1 in the regulation of bone mass. Liver IGF-1–deficient (LID) mice and acid labile subunit (a key component in the IGF-1 serum transport complex) knockout (ALSKO) mice have decreased serum IGF-1 levels and relatively normal bone. However, double-knockout LID × ALSKO (LA) mice are severely osteopenic.(11) A number of additional model systems further support a regulatory role for systemic IGF-1 in bone metabolism both as a direct regulator of osteoblast function and as a negative regulator of GH secretion.(12–14) Taken together, these results suggest that locally produced and circulating IGF-1 are important to skeletal growth and remodeling and are likely to have overlapping but not identical actions.

Adipocytes and osteoblasts are derived from bone marrow mesenchymal stromal cells.(15,16) Although cause and effect between increased bone marrow fat and osteoporosis has not been established with certainty, several lines of evidence suggest a relationship. A deficiency in PPARγ, a key mediator of adipocyte differentiation, reduces marrow fat and enhances osteogenesis.(17) Furthermore, a reciprocal relationship between bone mass and bone marrow adiposity has been noted frequently in humans and animal models.(18,19)

As indicated, GH secretion decreases with age. Also, senile and postmenopausal osteoporosis are associated with decreases in bone formation and increases in bone marrow adiposity. Studies investigating the effects of GH on bone marrow adiposity are few in number. In one such study, bone marrow adipocyte number and size were increased in the GH-deficient dwarf (*dw*) rat.(20) However, a more recent study using the same animal model detected a gender-specific increase in adipocyte number in females only.(21) Therefore, the purpose of this study was to clarify the role of GH signaling in regulating the balance between bone marrow fat and bone mass in severely GH-deficient hypophysectomized (HYPOX) female and male rats and in female and male mice with defective GH signaling.

## Materials and Methods

### Animals

Animals in the experiments described herein were maintained in accordance with the National Institutes of Health (NIH) *Guide for the Care and Use of Laboratory Animals,* and the experimental protocols were approved by the Institutional Animal Care and Use Committee.

HYPOX, ovariectomized (OVX), and sham-operated (control) female Sprague-Dawley rats (experiments 1 to 5) and HYPOX and sham-operated (control) male Sprague-Dawley rats (experiment 6) were obtained from Harlan (Indianapolis, IN, USA). The rats were housed individually in plastic shoebox cages in temperature- and humidity-controlled rooms with a 12/12 hour light/dark cycle. Rat chow and water were provided *ad libitum* to all animals.

Little (*lit*) mice (Experiment 7) on both a C57BL/6J (*lit/lit*) and a C3H/HeJ background (B6.C3H *lit/lit*) were raised at Jackson Laboratory (Bar Harbor, ME, USA) in the same temperature- and humidity-controlled rooms with a 14/10 hour light/dark cycle. LID mice (experiment 7) on a B6/129 mixed background were generated by Dr. Shoshana Yakar. The generation and housing of these mice were described previously.(22)

### Experimental design

#### Experiment 1

The effect of HYPOX on bone, bone marrow adiposity, and bone lipid content was determined in rapidly growing female rats. Four-week-old HYPOX (*n* = 10) and control (*n* = 5) Sprague-Dawley rats were used in this experiment because young rats are extremely sensitive to the growth-inhibitory effects of HYPOX. Fluorochrome labeling was used to determine longitudinal bone growth and mineralizing (double label) perimeter. Rats were injected subcutaneously (sc) with tetracycline (15 mg/kg; Sigma Chemical Co., St. Louis, MO, USA) 12 days prior to, calcein (15 mg/kg; Sigma) 4 days prior to, and demeclocycline (15 mg/kg; Sigma) 1 day prior to necropsy at 6 weeks of age. Blood was drawn immediately before necropsy for measurement of serum leptin and IGF-1 levels. Tibiae were harvested for histomorphometry and stored in 70% ethanol at 4°C prior to processing. Femora were frozen in liquid N_2_ and stored at −84°C prior to RNA and lipid analysis. Liver was frozen in liquid N_2_ and stored at −84°C prior to RNA analysis.

#### Experiment 2

This study was performed to determine the reversibility of HYPOX-induced skeletal abnormalities by GH replacement therapy. Sexually mature 3-month-old female rats were used in this and subsequent studies because older rats tolerate long-duration GH deficiency better than younger rats. One day before HYPOX, the animals received a perivascular tail injection of tetracycline (Sigma) at 20 mg/kg to label mineralizing bone matrix prior to treatment. The rats then were divided into five groups: (1) day 10 postoperative control (*n* = 9), (2) day 10 postop HYPOX (*n* = 11), (3) day 25 postop control + vehicke (VEH; *n* = 9), (4) day 25 postop HYPOX + VEH (*n* = 8), or (5) day 25 postop HYPOX + GH (*n* = 8). Starting on day 10 postoperatively, recombinant human GH (Genentech, San Francisco, CA, USA) was administered three times a day via sc injection at a dose of 800 µg/kg per day.

Because of the relatively long duration of this study, a daily substitution treatment with 500 mg/kg sc hydrocortisone (Solu Cortef, UpJohn, Kalamazoo, MI, USA) and 10 mg/kg sc thyroxine (T_4_, Sigma) was initiated in the HYPOX rats on the first postoperative day and continued for the 25 day duration of the experiment. This was done to exclude long-duration complications from HYPOX-associated hypothyroidism and corticosterone deficiency.(23) The pituitary-intact controls received daily sc saline injections. The rats were delivered to our facility overnight on postoperative day 7. On postoperative day 9, all rats received a 20 mg/kg perivascular tail injection of calcein (Sigma), and groups 1 and 2 were necropsied one day later. A third fluorochrome label, alizarin (20 mg/kg; Sigma) was administered on postoperative day 24, and the rats necropsied one day later.

#### Experiment 3

This study was performed to determine the potential contribution of serum 17β -estradiol (E_2_) or IGF-1 to bone marrow adiposity in HYPOX female rats. Thirty-four 3-month-old virgin female Sprague-Dawley rats received a sc perivascular tail injection of 20 mg/kg tetracycline. One day later, all animals underwent HYPOX. As in experiment 2, a daily substitution treatment with 500 mg/kg sc hydrocortisone and 10 mg/kg sc T_4_ was initiated on the first postoperative day and continued for the 25 day duration of the experiment. The rats were delivered to our facility overnight on postoperative day 7 and were divided into the following treatment groups: (1) HYPOX + VEH (saline, *n* = 8), (2) HYPOX + E_2_ (4.8 µg/kg 17β-estradiol, Sigma, via daily sc injection, *n* = 13), (3) HYPOX + GH (800 µg/kg/day recombinant human GH, Genentech, via three daily sc injections, *n* = 8), and (4) HYPOX + IGF-1 (long R3, 200 µg/kg/day, GroPep, Adelaide, Australia, via once-daily sc injection, *n* = 5).

Calcein (20 mg/kg) was administered to all rats on postoperative day 9, and baseline HYPOX controls were sacrificed the next day. Treatment with VEH, E_2_, GH, or IGF-1 was begun on postoperative day 10 and continued for 14 days. Alizarin (20 mg/kg) was administered on postoperative day 24, and the rats were necropsied the following day.

#### Experiment 4

This study was performed to confirm the lack of an effect of increased systemic IGF-1 levels on bone marrow adiposity in HYPOX female rats. The experimental design was the same as experiment 1 except that 3-month-old rats were used, and a group of HYPOX (*n* = 12) rats was infused continuously with recombinant human IGF-1 (GroPep) during the final 5 days of the study at a dose of 2,000 µg/kg per day using 7 day sc implanted osmotic pumps (Model 2001, Alzet, Cupertino, CA, USA).

#### Experiment 5

This study was performed to determine whether PTH decreases bone marrow adiposity in sexually mature HYPOX female rats. Sixteen 3-month-old virgin female HYPOX rats were used. The animals were supplemented with hydrocortisone and T_4_ as described in experiment 3. The rats were divided into three groups: (1) HYPOX + VEH (*n* = 5), (2) HYPOX + GH (800 µg/kg/day via three daily sc injections, *n* = 6), and (3) HYPOX + PTH [human PTH(1-34), 80 µg/kg/day, Bachem, Torrance, CA, USA, via once-daily sc injection, *n* = 5]. Treatment was started on postoperative day 5 and continued until sacrifice on postoperative day 19. Fluorochromes were administered on postoperative days 5 (calcein, 15 mg/kg) and 18 (alizarin, 15 mg/kg).

#### Experiment 6

This experiment was performed to determine the effects of HYPOX and GH replacement on bone histomorphometry and bone marrow adipocyte pool size in sexually mature 3-month-old male rats. The rats were divided into the following treatment groups: (1) control + VEH (*n* = 7), (2) HYPOX + VEH (*n* = 5), and (3) HYPOX + GH (recombinant human GH, Genentech, 800 µg/kg/day via twice-daily sc injection, *n* = 6). Treatment with VEH (saline) or GH was begun on postoperative day 10 and continued for 8 days. Demecloclycline (15 mg/kg) was administered 6 and calcein (15 mg/kg) 2 days prior to sacrifice, and the rats were necropsied on postoperative day 24. Following necropsy, tibiae were excised for histomorphometry, and bone marrow was harvested from femora of HYPOX and control rats for adipocyte culture.

#### Experiment 7

This study was performed to determine whether bone marrow adiposity is increased in *lit* or LID mice. Eight-week-old female *lit* (*n* = 11) and wild-type (WT, *n* = 12) littermates, as well LID (*n* = 4) and WT (*n* = 4) littermates, were used to evaluate the effects of GH insufficiency (GH levels in the *lit* mice are <5% of WT) and liver-derived IGF-1 deficiency in serum (LID mice) on marrow adiposity. Following necropsy, femora were excised for histomorphometry.

### Tissue collection

For tissue collection, all rats and mice were anesthetized with isoflurane or CO_2_. Death was induced by exsanguination from the heart, followed by cardiac excision. Serum was collected and stored at −20°C prior to analysis. Uteri were removed, blotted dry, and weighed. Livers and femora were collected from rats, frozen in liquid N_2_, and stored at −84°C for RNA analysis. Tibiae were excised from rats and femora from mice and placed in 70% ethanol for histologic processing.

### Serum measurements

Serum IGF-1 was measured with an RIA using a polyclonal antibody to IGF-1 after separation of IGF-binding proteins by acid ethanol extraction. The interassay coefficient of variation is 4.6%, and the lower limit of detection is 0.1 ng/mL.(24) Serum leptin was measured by ELISA as recommended by the manufacturer (Diagnostic Systems Laboratories, Webster, TX, USA, or R&D Systems, Minneapolis, MN, USA).

### Adipocyte culture

Primary bone marrow stromal cells were cultured in α modified essential medium (α-MEM) supplemented with 10% fetal bovine serum (FBS) and antibiotics at an initial density of 1, 2, and 4 × 10^6^ cells/mL in 10 cm^2^ dishes. After 10 days in culture, the cells were split and seeded at 8 × 10^6^ cells/mL in 6 well plates for induction of preadipocytes. A differentiation-inducing medium consisting of the base α-MEM–10% FBS supplemented with 1 µM Rosiglitazone, 1 µM dexamethasone, and 0.5 µM insulin was added for 7 days to allow mature adipocytes to develop. The cultures were fixed, and adipocytes were stained with oil red-O. The number of adipocytes per field was determined by counting adipocytes at 20× in eight randomly selected fields in 6 wells per treatment.

### Lipid analysis

The frozen right femora from 5 rats per group from experiment 1 were individually homogenized in a Spex Freezer Mill (Edison, NJ, USA). Total lipid was extracted from bone in chloroform-methanol (2:1) plus 1 mM butylated hydroxytoluene.(25) 7-Nonadecenoic acid (19:1, Nu-Chek Prep, Elysian, MN, USA) was added as a recovery standard at the time of extraction. Total triglyceride and cholesterol was measured using kits from Wako (Richmond, VA, USA). Lipid standards were provided by Wako. Total lipids were saponified, and fatty acids were fractionated and quantified by reverse-phase HPLC using a YMC J-Sphere (ODS-H80) column (Waters, Milford, MA, USA) and a gradient starting at 77.5% acetonitrile + acetic acid (0.1%) and ending at 99.9% acetonitrile + acetic acid (0.1%) over 90 minutes with a flow rate of 1.0 mL/min using a Waters 600 controller. Fatty acids were detected using both ultraviolet light absorbance at 192 nm (Model 2487, Waters) and evaporative light scatter (Model 2420, Waters). Fatty acid standards were obtained from Nu-Chek Prep (Elysian, MN, USA).

### Tissue processing

For histomorphometric evaluation of cancellous bone and marrow adiposity, proximal tibiae (rat) or distal femora (mice) were dehydrated in a graded series of ethanol and xylene and embedded undecalcified in modified methyl methacrylate, as described previously.(23,26) Longitudinal sections (4 µm thick) were cut with vertical-bed microtomes (Richert-Jung Supercut 2050 or Leica 2065, Bannockburn, IL, USA) and affixed to slides. One section per animal was stained according to the Von Kossa method with a tetrachrome counterstain (Polysciences, Warrington, PA, USA) or with toluidine blue and used for assessing bone marrow adiposity. A second section was left unstained and used for assessing fluorochrome-based measurements.

### Histomorphometry

Histomorphometric data were collected under visible or ultraviolet light using the OsteoMeasure System (OsteoMetrics, Inc., Atlanta, GA, USA). Cancellous bone and bone marrow adiposity were measured at a standardized site within the proximal tibial metaphysis in rats and the distal femoral metaphysis in mice. This site was located 1 mm distal to the growth plate in rats and 0.5 mm proximal to the growth plate in mice. Endpoints evaluated included bone area/tissue area (%), adipocyte area/tissue area (%), adipocyte number/tissue area (#/mm^2^), and adipocyte size (µm^2^). Adipocytes were identified morphologically using the following criteria: large circular or oval shape bordered by a prominent cell membrane and absence of cytoplasmic staining owing to alcohol extraction of intracellular lipids during processing. Mineralizing perimeter was measured as double-label (final two labels) perimeter/bone perimeter (%). In experiment 1, longitudinal growth rate in rats was calculated as the mean distance (determined at five different sites across the metaphysis) between the most proximal location of the calcein label and the distal end of the growth plate divided by 4 days (µm/day). All bone histomorphometric data are reported using standard nomenclature.(27)

### RNA isolation

#### Liver

Frozen liver samples were homogenized in TRIzol reagent (Invitrogen, Carlsbad, CA, USA). Total liver RNA was isolated according to the manufacturer's protocol. RNA quantity was determined spectrophotemetrically, and RNA quality was evaluated via formaldehyde agarose gel electrophoresis.

#### Bone

The frozen distal metaphysis was isolated from the left femur of 4 rats per group. Each metaphysis was individually homogenized with guanidine isothiocyanate in a Spex Freezer Mill (Edison, NJ, USA). Total cellular RNA was then extracted and isolated using an organic solvent method.(28) RNA yields were determined spectrophotometrically at 260 nm. 15 µg of RNA from each sample was then denatured by incubation at 52°C in a solution of 1 M glyoxal and 50% dimethylsulfoxide in 0.1 M NaH_2_PO_4_ and separated electrophoretically on a 1% agarose gel. The quality of RNA loaded on the gel was assessed as for liver.

### Real-time ploymerase chain reaction (PCR) and RNase protection assay for IGF-1

IGF-1 mRNA levels in the liver were measured using real-time PCR. cDNA for reverse transcriptase (RT)–PCR was synthesized using SuperScript First-Strand Synthesis System for RT-PCR (Invitrogen). One microgram of total RNA was reverse transcribed using random hexamer primers and SuperScript II reverse transcriptase according to manufacturer's protocol. Real-time PCR primers that are specific for rat IGF-1 (forward: 5'-CCGGACCAGAGACCCTTTG-3'; reverse: 5'-CCTGTGGGCTTGTTGAAGTAAAA-3') and rat 18S ribosomal RNA (18S) (forward: 5'-GGACCAGAGCGAAAGCATTTGC-3'; reverse: 5'- CGCCAGTCGGCATCGTTTATG-3') were synthesized by Operon Biotechnologies (Huntsville, AL, USA). IGF-1 and 18S real-time PCR reactions were performed using DyNAmo HS SYBR Green qPCR kit (New England Biolabs, Ipswich, MA, USA). A standard curve that was generated from serial dilutions of purified plasmid DNA that encoded the respective genes was used to measure mRNA transcript copy number. mRNA data represent normalized copy number of IGF-1 using the 18S housekeeping gene.

IGF-1 mRNA levels in bone were measured by RNase protection assay, as described previously.(29)

### Statistical analysis

The effects of treatment were analyzed using a one-way ANOVA followed by a Student-Newman-Keuls multiple-comparison test (SPSS 13.0, SPSS, Inc., Chicago, IL, USA). When the ANOVA assumptions of normality or homogeneity of variance were not met, a Kruskal-Wallis rank test followed by Dunn's multiple-comparison test was used. Differences were considered significant at *p* < .05. All data are reported as mean ± SE.

## Results

### Experiment 1

**Fig. 1:**
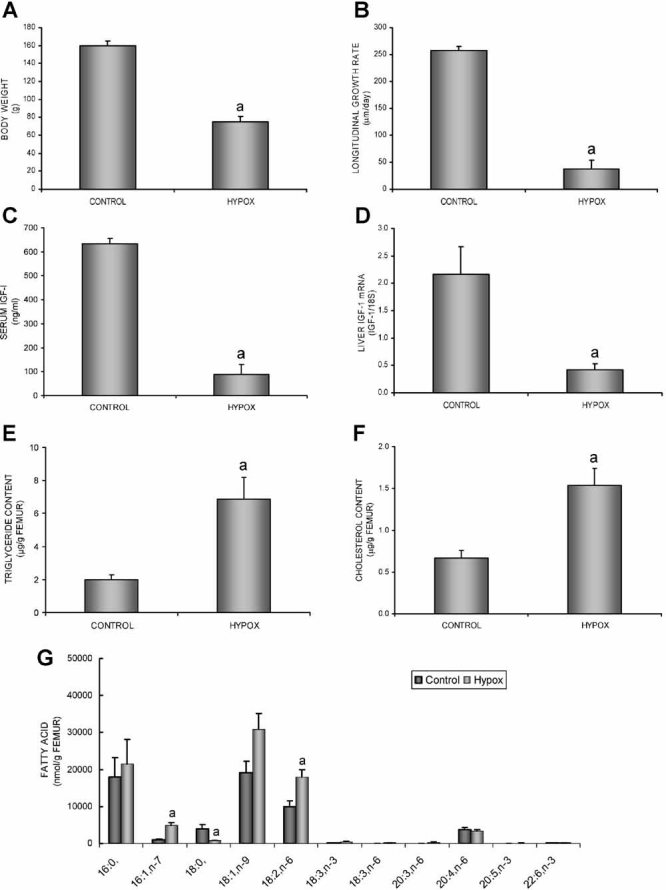
Effect of hypophysectomy (HYPOX) on body weight (*A*), longitudinal bone growth rate measured in the proximal tibia (*B*), serum insulin-like growth factor 1 (IGF-1) peptide (*C*), IGF-1 mRNA measured in liver (*D*), and triglyceride (*E*), cholesterol (*F*), and fatty acid composition (*G*) in total femur in weanling female rats (experiment 1). Values ± SE; *n* = 4 to 5 per group. ^a^Significantly different from control, *p* < .05.

The effect of HYPOX on weanling female rats is shown in Fig. 1. HYPOX rats were 53% lighter (see 1*A*) and had an 85% lower rate of longitudinal growth in the proximal tibia (see 1*B*) than age-matched controls. Serum IGF-1 was dramatically lower in HYPOX rats than in control rats (see 1*C*). mRNA levels for IGF-1 in the liver were likewise lower in HYPOX rats than in control rats (see 1*D*). HYPOX rats had higher triglyceride (see 1*E*) and cholesterol (see 1*F*) levels in bone compared than control rats. In addition, the fatty acid profile was altered in the HYPOX animals (see 1*G*); compared with controls, HYPOX rats had higher levels of 16:1,N-7 and 18:2,N-6 and lower levels of 18:0.

Representative photomicrographs of bone marrow from a control (2*A*) and a HYPOX (2*B*) rat clearly illustrate the dramatic increase in marrow adiposity following HYPOX. Compared with control rats, HYPOX rats had higher adipocyte number (see 2*C*) and size (see 2*D*), resulting in a greater percentage of marrow area occupied by adipocytes (see 2*E*). Mineralizing bone perimeter was drastically lower in HYPOX rats than in control rats (see 2*F*). Representative photomicrographs of fluorochrome labeling is shown for a control (2*G*) and HYPOX (2*H*) rat. Whereas the initial baseline label (tetracycline) was observed often in HYPOX animals, HYPOX resulted in a dramatic decrease in the final label (demeclocycline), suggesting a time-dependent decrease in mineralizing perimeter. Serum leptin levels averaged 2 ng/mL in control rats but were below the assay detection limit (0.5 ng/mL) in HYPOX animals (data not shown), suggesting a decrease in whole-body fat mass with HYPOX.

**Fig. 2:**
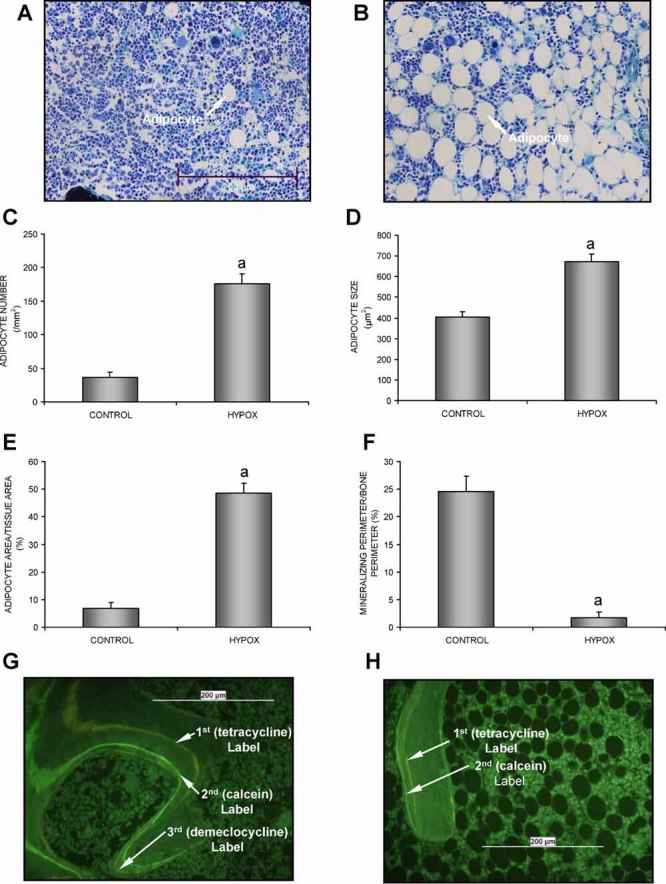
Effect of hypophysectomy (HYPOX) on bone marrow adiposity and mineralizing bone perimeter in weanling female rats (experiment 1). Representative micrographs show adipocytes in the proximal tibia of a control (*A*) and a HYPOX (*B*) rat. Note the increased adiposity in the HYPOX animal. Quantitative measurements were performed to obtain adipocyte number (*C*), adipocyte size (*D*), and adipocyte area/tissue area (*E*). Mineralizing perimeter (*F*) was measured, and representative micrographs show fluorochrome labels in a control (*G*) and a HYPOX (*H*) rat. Note the absence of the final (demeclocycline) label in the HYPOX rats. Values are mean ± SE; *n* = 5 per group. ^a^Significantly different from control, *p* < .01.

### Experiment 2

**Fig. 3:**
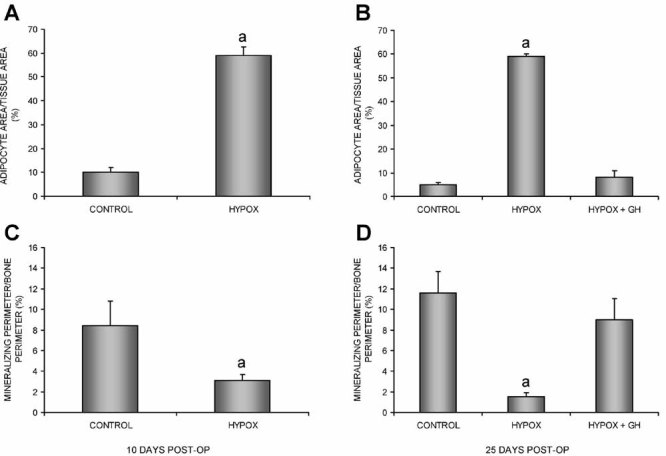
Reversibility of hypophysectomy (HYPOX)–induced skeletal abnormalities by GH in sexually mature female rats (experiment 2). The effects of HYPOX and GH replacement are shown for adipocyte area/tissue area (*A*, *B*) and mineralizing perimeter/bone perimeter (*C*, *D*). Adipocyte area was higher (*A*) and mineralizing perimeter was lower (*C*) in HYPOX compared with control rats at 10 days postoperatively. GH replacement was initiated in HYPOX rats on day 10 postoperatively. GH replacement normalized bone marrow adiposity (*B*) and mineralizing perimeter/bone perimeter (*D*) in HYPOX rats by postoperative day 25. Values are mean ± SE; *n* = 9 per group. ^a^Significantly different from control, *p* < .01.

The effects of HYPOX and GH replacement on bone marrow adiposity and mineralizing perimeter in sexually mature female rats are shown in Fig. 3. Bone marrow adiposity was much higher in HYPOX animals than in control animals on day 10 postoperatively (see 3*A*) and remained higher on postoperative day 25 (see 3*B*). GH replacement, starting on day 10 postoperatively, reduced bone marrow adiposity to near sham levels by postoperative day 25 (see 3*B*). Mineralizing perimeter was decreased during the initial 10 days following HYPOX (see 3*C*) and remained lower at 25 days postoperatively (see 3*D*). GH replacement starting on postoperative day 10 restored mineralizing perimeter to near the age-matched control level by postoperative day 25 (see 3*D*).

### Experiment 3

**Fig. 4:**
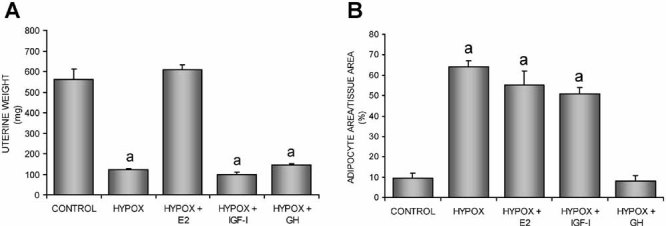
Effect of treatment with 17β-estradiol (E_2_), insulin-like growth factor 1 (IGF-1), or growth hormone (GH) on uterine weight (*A*) and bone marrow adipocyte area/tissue area in the proximal tibia (*B*) in sexually mature hypophysectomized (HYPOX) female rats (experiment 3). Hormone replacement was started 10 days following HYPOX. Values are mean ± SE; *n* = 8 per group). ^a^Significantly different from control, *p* < .01.

The effects of HYPOX and treatment with E_2_, IGF-1, or GH on uterine weight and bone marrow adiposity in sexually mature female rats are shown in Fig. 4. Uterine weight was reduced dramatically by HYPOX, increased in HYPOX rats following E_2_ replacement, but unchanged in HYPOX rats treated with IGF-1 or GH (see 4*A*). HYPOX increased bone marrow adiposity (see Fig. 4B). Treatment with GH decreased bone marrow adiposity following HYPOX. In contrast, treatment with E_2_ or IGF-1 had no effect on bone marrow adiposity in HYPOX rats.

### Experiment 4

As was the case for intermittent IGF-1 (experiment 3), continuous infusion of IGF-1 (2,000 µg/kg/day) had no effect on bone marrow adiposity. Compared with control rats (5.4 ± 1.4, mean ± SE), adipocyte area was much greater in HYPOX (53.4 ± 3.7, *p* < .01) and IGF-1-treated HYPOX (49.2 ± 3.1, *p* < .01) rats. The latter two groups did not differ from one another.

### Experiment 5

**Fig. 5:**
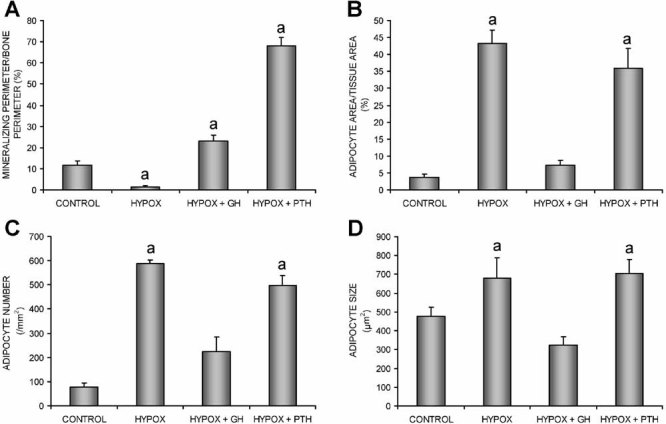
Effect of treatment with growth hormone (GH) or intermittent parathyroid hormone (PTH) on mineralizing perimeter/bone perimeter (*A*), bone marrow adipocyte area/tissue area (*B*), adipocyte number (*C*), and adipocyte size (*D*) in the proximal tibia in sexually mature hypophysectomized (HYPOX) female rats (experiment 5). Hormone replacement was started 10 days following HYPOX. Note that PTH increased mineralizing perimeter but in contrast to GH had no effect on bone marrow adiposity. Values are mean ± SE; *n* = 5 to 6 per group. ^a^Significantly different from control, *p* < .01.

The effects of HYPOX and treatment with GH or PTH on mineralizing perimeter and bone marrow adiposity in sexually mature female rats are shown in Fig. 5. HYPOX resulted in a decrease in mineralizing perimeter that was reversed by GH and PTH (see 5*A*). Administration of GH to HYPOX rats also decreased adipocyte area, number, and size (see Fig. 5*B–D*). In contrast, PTH had no effect on bone marrow adiposity in the HYPOX animals. mRNA levels for IGF-1 in the distal femur were significantly higher in control (169 ± 6%) and HYPOX rats treated with GH (366 ± 134%) or PTH (351 ± 13%) compared with HYPOX rats (100 ± 6%).

### Experiment 6

**Fig. 6:**
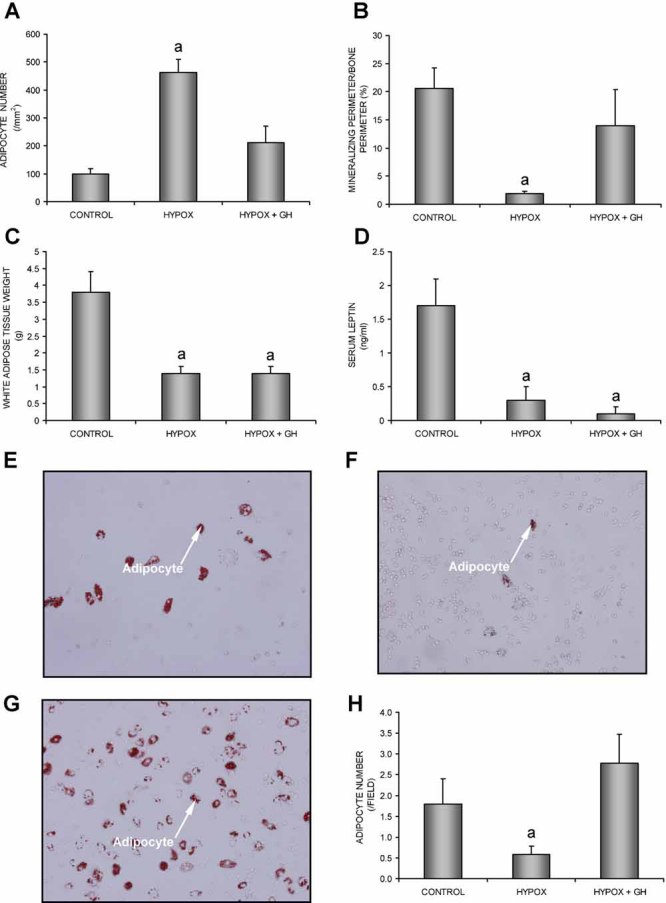
Effect of hypophysectomy (HYPOX) and growth hormone (GH) replacement on adipocyte number (*A*), mineralizing perimeter/bone perimeter (*B*), abdominal white adipose tissue (WAT) weight (*C*), and serum leptin (*D*) in sexually mature male rats (experiment 6). Values are mean ± SE; *n* = 5 to 7 per group. ^a^Significantly different from control, *p* < .01. Also shown are representative micrographs indicating adipocytes in cell culture from control (*E*), HYPOX (*F*), and HYPOX + GH (*G*) rats and adipocyte number/field (*H*). Values are mean ± SE; *n* = 6 replicates per group. ^a^Significantly different from control, *p* < .05.

The effects of HYPOX and treatment with GH in sexually mature male rats in vivo and in bone marrow–derived cell culture from each respective group of rats are shown in Fig. 6.

#### In vivo

Compared with control, HYPOX resulted in higher adipocyte numbers in bone marrow (see 6*A*) but lower mineralizing perimeter (see 6*B*), white adipose tissue (WAT; see 6*C*), and serum leptin (see 6*D*) levels in male rats. Short-duration (8 day) GH treatment resulted in lower bone marrow adiposity and higher mineralizing perimeter compared with HYPOX. Differences in WAT or serum leptin levels were not observed with the GH treatment.

#### In vitro

Representative oil red O–stained preadipocyte cultures from control, HYPOX, and HYPOX + GH–treated rats are shown in Fig. 6*E–G*, respectively. HYPOX reduced and GH replacement of HYPOX rats normalized the number of adipocytes generated during bone marrow culture (see 6*H*).

### Experiment 7

**Fig. 7:**
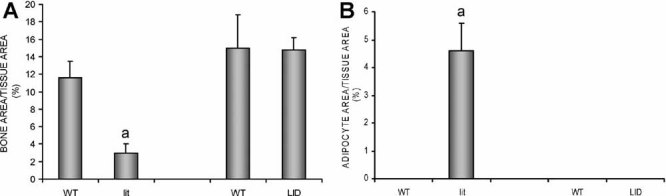
Bone area and marrow adiposity in genetic models of impaired growth hormone (GH) signaling. Bone area/tissue area (*A*) and bone marrow adipocyte area/tissue area (*B*) in *lit* and LID mice and their respective wild-type (WT) littermates (experiment 7). Values are mean ± SE; *n* = 11 (*lit* and littermate control) or *n* = 4 (LID and littermate control). ^a^Significantly different from WT, *p* < .01.

Cancellous bone area and adipocyte area are shown for female *lit* and LID mice in Fig. 7. *lit* mice were severely osteopenic (see 7*A*) and had greater bone marrow adiposity than WT mice (see 7*B*). Data are shown for *lit* mice on a C57BL/6J genetic background. Similar results were obtained for *lit* mice on a C3.B6 background (data not shown). Gender had no effect on either cancellous bone area or bone marrow adiposity in these mice (data not shown).

In contrast to *lit* mice, LID mice did not differ from WT mice in cancellous bone area/tissue area (see 7*A*) or bone marrow adiposity (see 7*B*).

## Discussion

HYPOX decreased weight gain and longitudinal bone growth. Cancellous bone area and mineralizing bone perimeter also were decreased following HYPOX, whereas marrow adiposity was increased. Increased bone marrow adiposity with HYPOX was detected in rapidly growing female as well as sexually mature female and male rats. Serum IGF-1 levels plummeted following HYPOX, as did mRNA levels for IGF-1 in liver and bone. The rats also developed hypoleptinemia that was not reversed following short-duration GH replacement. Administration of GH reversed the increased bone marrow adiposity associated with HYPOX prior to changes in WAT or serum leptin levels. Mineralizing perimeter, an index of active osteoblasts, was increased by GH or PTH in HYPOX rats. In contrast, treatment of HYPOX rats with hydrocortisone and T_4_, with or without E_2_ or IGF-1, was ineffective in preventing marrow adiposity in these animals. Compared with WT mice, GH-deficient *lit* mice had increased bone marrow adiposity, whereas bone marrow adiposity in IGF-1-deficient LID mice was normal.

GH deficiency in HYPOX male and female rats and female *dw* rats is accompanied by truncal leanness and hypoleptinemia.(21) Tibial marrow adiposity was reported to be normal in male but higher in female *dw* rats. The gender-dependent difference in bone marrow adiposity in females was attributed to higher adipocyte number. The more dramatic increase in bone marrow adiposity associated with complete GH depletion owing to HYPOX observed in both sexes in the current studies was due to a combination of increases in the number and size of the adipocytes and was accompanied by increases in femoral triglycerides and cholesterol. Thus the precise presentation of skeletal phenotype in the various models described herein may be dependent on species, strain, or magnitude of GH deficiency. In this regard, studies performed in the *oMtla*-*oGH* transgenic mouse model suggest that the level of GH receptor and IGF-1 expression act through a threshold mechanism.(30)

HYPOX resulted in changes in the fatty acid profile of bone. There was a shift in 16- and 18-carbon fatty acids to become more unsaturated. This almost certainly reflects an increase in bone marrow stearoyl CoA desaturase (SCD1) activity following HYPOX. This finding is consistent with the reported inhibition by GH of *SCD1* gene expression in adipose tissue.(31) Increases in SCD1 are associated with adiposity,(32) and fatty acids are known to regulate adipocyte differentiation and metabolism,(33) providing a potential mechanism for the increase in marrow adiposity with GH deficiency.

Our studies show that HYPOX decreases and GH increases the size of the preadipocyte pool in the bone marrow of male rats. These results are in agreement with a previous study.(34) Other studies suggest that GH inhibits expression of late genes required for terminal adipocyte differentiation.(35) Thus the increased adipocyte number following HYPOX in vivo may be due to an increase in differentiation of cells already committed to become adipocytes. These observations are consistent with evidence that GH enhances the lipolytic activity of adipocytes(36) and inhibits their lipoprotein lipase activity.(37) As a result, GH deficiency would be expected to enhance triglyceride accumulation by adipocytes. These observations support the hypothesis that GH increases adipocyte as well as osteoblast precursor populations in bone marrow but also suppresses fat accumulation. If this interpretation is correct, GH signaling plays a critical role in establishing the balance between bone and fat mass.

HYPOX prevents the synthesis of luteinizing hormone, follicle-stimulating hormone, thyroid-stimulating hormone, adrenocorticotrophic hormone, α-melanocyte-stimulating hormone, antidiuretic hormone, oxytocin, prolactin, and GH. HYPOX indirectly alters the levels of additional hormones and growth factors known to influence bone growth and turnover. These include IGF-1, gonadal hormones (E_2_), adrenal hormones (corticosterone), thyroid hormones (thyroxine), and adipokines (leptin).(38–42) GH replacement normalizes some (e.g., IGF-1) of these factors. Therefore, the effects of GH replacement on bone and marrow fat in HYPOX animals could be indirect.

HYPOX results in decreases in serum gonadal hormones to values comparable with those observed following OVX.(43) The effect of OVX to reduce cancellous bone mass in the rat is well established.(44) Also, bone marrow adiposity is increased in rats following OVX.(45) For these reasons, we investigated the contribution of estrogen deficiency to bone marrow adiposity by treating HYPOX animals with E_2_. As expected, E_2_ treatment increased uterine weight in the HYPOX rats.(38,40) However, E_2_ had no effect on bone marrow adiposity. This finding suggests that GH deficiency rather than estrogen deficiency is responsible for the rapid increase in bone marrow adiposity following HYPOX.

T_4_ is reported to influence fetal preadipocyte development and to enhance IGF-1 secretion by adipocytes in cell culture.(43) Fetal HYPOX in pigs slightly enhanced lipid accretion in adipose tissue and markedly increased fat cell size and de novo lipogenesis. T_4_ treatment had no influence on fat cell size but markedly increased lipogenesis, lipid accretion, and adipocyte number in HYPOX fetuses.(46) In our longer-duration studies, HYPOX and GH-treated female rats were supplemented with T_4_. T_4_ supplementation did not prevent the reduction of bone marrow adiposity in GH-treated HYPOX rats. Also, we have performed shorter-duration studies in HYPOX rats not supplemented with either T_4_ or hydrocortisone that document similarly increased adiposity following HYPOX and reversal of bone marrow adiposity in HYPOX rats by GH.

IGF-1 is required for GH-regulated bone growth and turnover.(12–14) As discussed previously, the actions of GH could be mediated by systemic and/or locally produced IGF-1. The principal source of the former is the liver. In this study, the precipitous decrease in circulating levels of IGF-1 following HYPOX was associated with decreased liver mRNA levels for the growth factor. This finding is in agreement with previous results.(47,48) The observed decrease in mRNA levels for IGF-1 in the distal femur provides evidence that HYPOX reduces the local skeletal production of IGF-1. Thus the observed efficacy of GH replacement to reverse the effects of HYPOX on bone and fat could be mediated by systemic and/or locally produced IGF-1.

Administration of intermittent or continuous IGF-1 to HYPOX rats did not prevent the skeletal effects of HYPOX, a finding that is in agreement with studies in *dw* rats.(20) However, none of the studies to date excludes the possibility that the administered IGF-1 was not bioavailable to osteoblasts and adipocytes. Therefore, we investigated genetic models of impaired GH signaling. *lit* mice have low GH levels owing to an autosomal recessive mutation for the growth hormone–releasing hormone receptor.(49) As is the case with HYPOX and *dw* rats, *lit* mice have very low serum IGF-1 levels. As shown in this study, *lit* mice have very low cancellous bone mass and elevated bone marrow adiposity. LID mice, on the other hand, have low circulating IGF-1 but elevated GH levels.(50) Thus, not only did IGF-1 replacement not reduce bone marrow adiposity in HYPOX and GH-deficient *dw* rats, but a congenital low circulating level of IGF-1 also did not induce bone marrow adiposity in GH-replete mice. Additionally, we have evaluated two other mouse strains with reduced IGF-1 levels, the acid labile subunit knockout (ALSKO) and the LID × ALSKO (LA) knockout. The LA strain has exceptionally low serum IGF-1 levels. As was the case for LID mice, neither ALSKO nor LA mice had increased bone marrow adiposity (data not shown). Taken as a whole, findings presented here suggest that GH may be acting on bone and fat mass largely through GH receptors on osteoblast and adipocyte lineage cells.

The effects of GH on osteoblasts and bone marrow–derived adipocytes may be mediated through locally produced IGF-1. Several lines of evidence implicate a requirement of locally produced IGF-1 in osteoblast lineage cells for the bone anabolic response to PTH.(50,51) As a consequence, PTH can bypass GH signaling and stimulate bone formation in HYPOX rats.(23) In this report, PTH increased mRNA levels for IGF-1 and bone mineralizing perimeter in long bones of HYPOX rats but did not prevent the increase in bone marrow adiposity associated with GH deficiency. These findings do not support a cause-and-effect relationship between increased bone marrow adiposity and decreased bone formation and clearly disassociate the inhibitory effects of GH on bone marrow adiposity from the hormone's stimulatory effect on bone formation.

HYPOX resulted in profound decreases in WAT and serum leptin levels. Leptin, an adipocyte-derived hormone, is believed to have direct and indirect effects on bone formation,(52) adipocyte differentiation,(53) and GH secretion.(54) Therefore, it is possible that some of the skeletal effects observed following HYPOX are due to leptin deficiency. Davies and colleagues(21) observed increased blood leptin levels 14 days following initiating treatment of GH-deficient *dw* rats with GH. However, in these studies we have shown that GH replacement increases mineralizing bone perimeter and decreases adipocyte number and size prior to having an effect on WAT weight or serum leptin levels. The present findings do not exclude a contributing role for leptin but clearly demonstrate the important role of GH in regulating bone and fat metabolism.

In summary, we conclude that GH deficiency is responsible for the reductions in bone mineralizing perimeter and increases in bone marrow adiposity in HYPOX rats and *lit* mice. However, the increase in bone marrow adiposity following HYPOX is unlikely to be directly responsible for decreased bone formation. Thus these and other studies(55,56) suggest that changes in osteoblast differentiation need not be directly coupled to changes in adipocyte differentiation. Further studies are needed to determine whether reduced GH signaling is responsible for the age-related reciprocal changes in bone and fat.
